# Design, Calibration and Characterization of a Fiber Optic Triaxial Accelerometer Based on Fiber Bragg Gratings

**DOI:** 10.3390/s26051588

**Published:** 2026-03-03

**Authors:** Roney Duarte da Silva, João Marcos Salvi Sakamoto

**Affiliations:** 1 Graduate Program in Science and Space Technologies, Aeronautics Institute of Technology (ITA), Sao Jose dos Campos 12228-900, SP, Brazil; roneyddasilva@gmail.com; 2 Division of Photonics, Institute for Advanced Studies (IEAv), Sao Jose dos Campos 12228-001, SP, Brazil

**Keywords:** fiber Bragg grating (FBG), optical accelerometer, temperature calibration, inertial navigation, optomechanical design

## Abstract

This work presents the design, calibration and detailed performance characterization of a triaxial accelerometer based on fiber Bragg gratings (FBG), intended for space navigation applications. The sensor employs a single seismic mass architecture, whose acceleration-induced displacement deforms six optical fibers (OFs), forming twelve fiber segments (FSs) that act as elastic elements, with the strain measured by FBGs inscribed in each fiber. The methodology ranges from the manufacturing and spectral characterization of the FBGs to the design of a differential optical interrogation system and a low-noise signal conditioning circuit. A cornerstone of this work is the proposal of an extended calibration model that, in addition to the conventional sensitivity matrix and bias vector parameters, incorporates polynomial terms to actively compensate for the effects of temperature variation. This model was validated through tests in a climatic chamber, subjecting the sensor to different orientations and controlled temperatures. The experimental results validate the design’s effectiveness, demonstrating that the accelerometer achieves tactical-grade performance with a bias instability below $1.9\;mg_{E}$ for all axes. The analysis confirmed that the sensor’s effective full-scale range is approximately $\pm 20g_{E}$, and sensitivity of 112 pm/$g_{E}$, limited by the nature of the optical interrogation system. Furthermore, a third-order polynomial thermal compensation model was shown to provide the most efficient balance between model complexity and error reduction, reducing errors to a level dominated by the system’s intrinsic noise and ensuring the sensor’s accuracy over a wide operational temperature range.

## 1. Introduction

In aerospace and defense environments, autonomy and reliability are critical, particularly in scenarios where access to Global Navigation Satellite System (GNSS) signals may be denied, jammed or spoofed (which may occur in conflict areas), intermittent or degraded (such as in urban areas, indoors, near thick forests or canyons) [1,2,3]. Furthermore, GNSS signals may be entirely unavailable, as is in planetary and deep-space exploration, underwater or underground regions [4].

Proper navigation in such scenarios can be achieved through an inertial navigation system (INS). Consequently, the development of high-performance inertial sensors is a cornerstone for advancing navigation, guidance, and control systems, being of strategic importance in the aerospace and defense sectors. The primary inertial sensors composing an INS are the gyroscope and the accelerometer, the latter being the scope of this work. Fundamentally, accelerometers for navigation applications operate as mass-spring-damper systems, in which the deformation of an elastic element suspending a seismic mass is measured. Relative displacement of the mass is induced by applied acceleration, and the precise measurement of this deformation constitutes the operating principle of these devices.

Although conventional technologies, such as piezoelectric accelerometers and those based on micro-electro-mechanical systems (MEMS), represent mature solutions in the aerospace industry, limitations are presented in specific scenarios. For instance, susceptibility to electromagnetic interference (EMI) and ground loops is observed in piezoelectric sensors, necessitating shielded cables and local signal conditioning that increase weight and system complexity. In contrast, critical advantages for harsh environments are offered by fiber Bragg gratings (FBG)-based accelerometers: intrinsic immunity to EMI, resistance to chemical corrosion, and long-distance transmission capabilities enable operation near engines and high-power actuators without the requirement for complex shielding [5]. Moreover, a notable disadvantage of conventional electrical systems is the point-to-point cabling topology. This limitation is overcome by the multiplexing capability of FBGs, which enables the interrogation of dozens of sensors on a single optical fiber bus, significantly reducing cabling mass and volume [6].

FBGs have emerged as a promising technological alternative, functioning as selective spectral filters fabricated within the core of an optical fiber (OF). The wavelength reflected by the grating is shifted by mechanical strain or temperature variations applied to the fiber, enabling the correlation of this spectral shift with the physical quantity of interest [6]. The earliest developments of FBG-based accelerometers date back to the 1990s, when the feasibility of the concept was first demonstrated. In 1996, an initial prototype was presented wherein a sensitivity of $1\;{mg}_{E}/\sqrt{\mathrm{Hz}}$ with a flat frequency response up to 1 kHz was achieved by the strain induced in an FBG by a 10 g mass [7]. The unit $g_{E}$ represents the standard acceleration of Earth’s gravity, defined as $1\;g_{E}\approx 9.81\;m/s^{2}$. The process of converting the FBG wavelength variation into a measurable electrical signal, termed optical interrogation, was also explored during this period. An accelerometer designed for high-magnitude impulse measurement was described, employing an intensity-based interrogation method in which a laser is tuned to the edge of the FBG’s reflection spectrum [8].

Since these foundational works, the technology has evolved considerably, particularly concerning ingenious structural topologies [9,10,11,12,13,14,15,16,17,18,19,20,21,22]. Although the spring-mass topology remains prevalent due to its mechanical robustness, alternative configurations have been explored [23]. Recent studies have reported sensors based on multi-core fibers [24], as well as off-axis FBGs and flexible filament-based designs capable of multidimensional vibration sensing without bulky seismic masses, as reviewed by Guo et al. [5].

Significant improvements in sensitivity have been observed. An integrated single-mass triaxial design was investigated by Liu et al. [13], demonstrating superior consistency between axis sensitivities (98 pm/$g_{E}$ to 217 pm/$g_{E}$) and low cross-axis error (<9.5%). A low-frequency (1 Hz to 40 Hz) dual-mass sensor was proposed by Qiu et al. [22], achieving high sensitivity (1194 pm/$g_{E}$) and linearity ($R^{2}=99.98\%$), making it suitable for infrastructure monitoring. Beyond structural topology, recent research has also focused on manufacturing materials and cross-sensitivity decoupling. Low-frequency vibration sensors integrating FBGs into 3D-printed bridge-type flexure hinges were developed by Velázquez-Carreón et al. [11]. It was demonstrated that varying geometric parameters enables tuning of the dynamic response, achieving sensitivities up to 1730 pm/$g_{E}$ in the 1 Hz to 20 Hz range. Regarding seismic applications, a cross-diaphragm structure optimized for the 0.1 Hz to 50 Hz range with a sensitivity of 590 pm/$g_{E}$ was developed by Zhang et al. [21], while a five-fold sensitivity increase (≈$2150\;pm/g_{E}$) was achieved by Chen et al. [25] through the utilization of fibers with reduced cladding diameter (50 μm), validating a dynamic resolution of 0.1 m$g_{E}$.

While significant advancements in vector FBG accelerometers for seismic and structural applications have been reported in the recent literature—such as the integrated single inertial body sensor by Liu et al. [13] or the ultra-low frequency (0.05 Hz) device by Qiu et al. [12]—distinct challenges are imposed by inertial navigation applications. Precise three-dimensional vector measurement [23], as well as a frequency response extending to DC (0 Hz) for constant gravity vector monitoring, are required by navigation systems. This capability gap remains a challenge for many compact FBGs sensors, which are often limited to AC components (>0 Hz) due to the interrogation method or their piezoelectric nature.

Furthermore, to ensure the metrological reliability required for high-performance navigation, it is established that the characterization of accelerometers must follow standardized guidelines, such as the IEEE Std 1293-2018 [26]. While sensitivity is optimized in the aforementioned studies, a critical and often overlooked aspect is the rigorous characterization of thermal bias stability under these standardized protocols. It is noted in inertial navigation theory that bias stability is the governing parameter during the initial alignment and ‘warm-up’ phases, determining the accuracy of attitude estimation while the vehicle is stationary.

A prevalent difficulty in the design of FBG accelerometers is the cross-sensitivity distinction between strain and temperature. A common approach to mitigate this is the use of a differential arrangement [27,28,29]. Another strategy involves decoupling temperature from strain, as proposed by Huang et al. [30], utilizing chirped fiber Bragg gratings (CFBG) encapsulated in glass fiber-reinforced polymer. This method successfully decoupled strain from temperature by exploiting the distinct responses of the central wavelength and the full width at half maximum of the FBG peak (FWHM). However, unlike the intensity-based approach adopted in differential topologies, monitoring the central wavelength response requires the acquisition of the full spectral profile via a high-resolution optical spectrum analyser (OSA). Consequently, deployment in compact, high-speed embedded systems is limited by this requirement. Furthermore, the cost and volume of commercial interrogators remain significantly higher than those of integrated MEMSs electronics, the extreme miniaturization of which remains unmatched for applications with severe space constraints. In this context, the proposal of a custom, simplified, and low-cost interrogation system based on intensity modulation, designed to be easily embedded in a launch vehicle payload, constitutes the first contribution of this work.

Although the differential topology mitigates effects on the sensing element, and the decoupling of temperature from strain is a significant step forward, residual errors persist. This occurs because not only is the FBG subjected to temperature variations, but the complete accelerometer system, including peripheral optical components, remains susceptible to thermal fluctuations. The response of passive components, such as optical couplers, exhibits temperature dependence [31,32]. Similarly, power and wavelength fluctuations are displayed by the optical source (super-luminescent diode (SLD)) [33]. These systemic instabilities manifest as bias and scale factor errors, underscoring the need for a calibration model that actively compensates for the thermal dependence of the entire measurement chain. A developed reference method is employed for the triaxial sensor calibration, building upon the core methodology established by Kuncar et al. [34] and previously implemented and validated on embedded hardware in da Silva et al. [35]. Simultaneous and complete adjustment of the scale factor matrix and the misalignments relative to a reference field is enabled by this method. However, it was revealed by experimental tests that, at the system level, differential interrogation alone is insufficient to compensate for the aforementioned thermal effects. Therefore, the proposal of an extended polynomial calibration model represents the second contribution of this work. The thermal compensation of the photodetector, the SLD light source, and the optical couplers is fundamentally integrated by this model as a “black box” system-level method, effectively correcting the aggregate non-linearities of the optoelectronic chain.

Another critical and often overlooked aspect in these developments is the rigorous characterization of thermal dependence and long-term instability. As previously discussed, bias stability is the governing parameter for the initial alignment of navigation systems. Addressing this gap, a rigorous characterization of the sensor’s thermal dependence and long-term instability using Allan Variance analysis [36] is presented as the third contribution of this work. The sensor was tested in a climatic chamber, where a bias instability below $2mg_{E}$ was achieved, validating the proposed architecture and calibration methodology for tactical-grade applications.

In summary, to address the identified gaps—specifically the lack of system-level thermal compensation and the absence of rigorous stochastic noise quantification for navigation—the main contributions of this study are consolidated as follows:

1. Embedded Intensity-Based Interrogation: Development of a custom, low-cost, and compact interrogation system that enables DC-coupled acceleration measurements, overcoming the size, weight, and power (SWaP) limitations of commercial spectral interrogators.
2. System-Level Calibration Model: Proposal of an extended polynomial calibration methodology that compensates for the aggregate thermal non-linearities of the entire optoelectronic chain (source, couplers, and detectors), surpassing the limitations of transducer-only compensation.
3. Rigorous Stability Analysis: A comprehensive characterization of the sensor’s long-term bias instability and velocity random walk (VRW) using Allan Variance, validating its performance against tactical-grade navigation requirements.

The remainder of this paper is organized as follows. The accelerometer concept and its operating principle are described in Section 2. The design and fabrication methodology are detailed in Section 3. Experimental results are presented and discussed in Section 4. Finally, the conclusions of this work are summarized in Section 5.

## 2. Accelerometer Concept

The operating principle of an FBG-based accelerometer is the dynamic detection of shifts in the reflected spectrum, caused by strain on the OF [5]. Conceptually, the device operates as a mass-spring-damper system, in which a seismic mass is suspended by elastic elements. The acceleration applied to the sensor induces a relative displacement of the mass, and the measurement of the resulting strain in the elastic elements constitutes the measurement principle. In this design, segments of OF act as the elastic elements, and the strain is measured by the embedded FBGs.

Triaxial accelerometers are described in the literature in two main topologies: the modular one, which consists of three orthogonally mounted one-dimensional sensors, and the single seismic mass one. In this work, the second topology is adopted, in which the spatial displacement of the seismic mass induces strain in a set of fiber segments (FSs) containing FBGs, and this strain encodes the information about the three-dimensional components of acceleration [5].

The strain transducing element is the fiber Bragg gratings (FBG), an optical component consisting of an OF whose core refractive index has been periodically modulated along its axis. This structure acts as a selective spectral filter, reflecting a narrow band of wavelengths, the peak of which is the Bragg wavelength ($\lambda_{g}$). When the fiber is subjected to mechanical strain (ε) or a temperature variation (ΔT), the grating period changes, causing a shift in the reflected wavelength. This spectral variation ($\Delta \lambda_{g}$) is modeled by the Bragg equation [6]:

(1) $$\frac{\Delta \lambda_{g}}{\lambda_{g}}=\left(1-p_{e}\right)\varepsilon +\left(\alpha +\zeta \right)\Delta T.$$

The effective photo-elastic constant, $p_{e}$, is approximately 0.21, while the thermal expansion, α, and thermo-optic, ζ, coefficients are $0.55\;\times \;10^{-6}/{}^{\circ}C$ and $8.6\;\times \;10^{-6}/{}^{\circ}C$, respectively [6,37]. In the context of the accelerometer, the applied acceleration deforms the FSs, and by monitoring the variation in $\lambda_{g}$, the acceleration that caused it is inferred. The mechanical strain ε in the section of the OF that contains the FBG is expressed as a function of the initial length $\ell_{0}$ $\left[m\right]$ and the final length *ℓ* $\left[m\right]$, as follows:

(2) $$\varepsilon =\frac{\ell -\ell_{0}}{\ell_{0}}=\frac{\Delta \ell }{\ell_{0}}.$$

**Figure 1:**
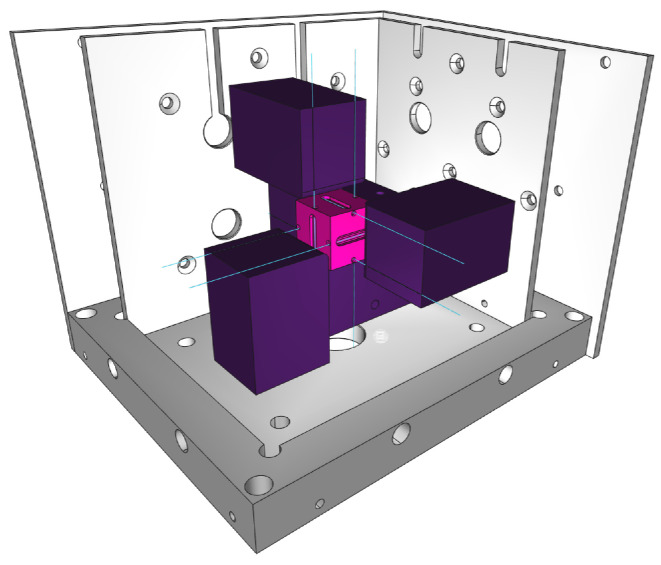
Three-dimensional CAD rendering of the AOM assembly. The internal seismic mass (pink) is suspended from the outer fixed frame (purple supports) solely by the OFs (blue lines). The mounting points on the base ($b_{n}$) and on the mass ($m_{n}$) define the active sensing segments where the FBGs are located.

**Figure 2:**
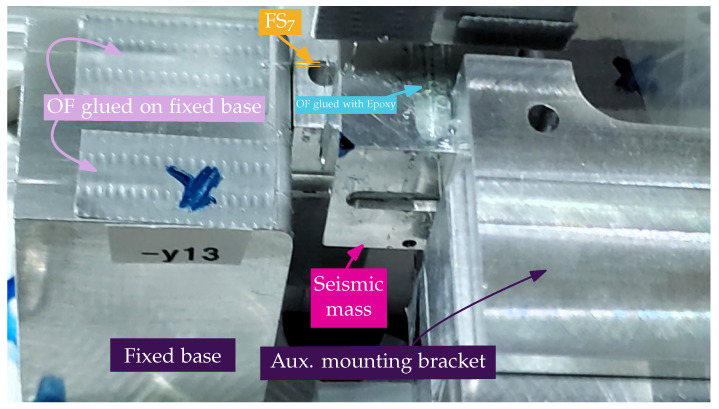
Photograph of the accelerometer during the assembly phase. The central aluminum seismic mass (magenta arrow) is positioned between the fixed base blocks (purple labels). The optical fibers are aligned and glued to the fixed base grooves. The yellow markers highlight the specific fiber segment ${\mathrm{FS}}_{7}$ suspended between the base and the mass before the final epoxy curing. The region indicated in cyan shows the gap where the fiber is bonded to the seismic mass.

The proposed sensor is a modified version of the concept by Cazo [28], as shown in the schematic model in Figure 1. The sensor’s base (purple supports) is mechanically coupled to the body under measurement, while the seismic mass (pink inner cube) responds to applied accelerations by modifying the tension in the FSs. This relative movement stores elastic potential energy in the fibers, which constitutes the measurement principle. To visualize the practical realization of this concept, Figure 2 presents a photograph of the sensor during the assembly phase, highlighting the interaction between the mechanical structure and the optical fibers.

A crucial distinction must be made between the physical OF and the functional FS. As observed in Figure 2, the OF is a continuous glass strand installed on the structure. However, this fiber is rigidly attached to the seismic mass using epoxy resin at points denoted by $m_{n}$ (highlighted in cyan), and to the fixed base at points $b_{n}$ (highlighted in light magenta). Note that, throughout this manuscript, bold mathematical symbols denote vectors. Mechanically, the specific section of fiber spanning between a base point $b_{n}$ and its corresponding mass point $m_{n}$ acts as an isolated elastic element. Therefore, for mechanical analysis, this active span is termed FS *n* (or ${\mathrm{FS}}_{n}$), effectively behaving as an individual spring element whose strain is measured via an inscribed FBG. Twelve such fiber segments are implemented in this design, four per axis.

Conversely, from the perspective of the optical interrogation circuit, the fiber paths remain optically continuous. As seen in the assembly photo, the light entering at point $b_{1}$ travels through the segment ${\mathrm{FS}}_{1}$, loops around, and exits through the opposing segment (e.g., at point $b_{3}$). Thus, one continuous OF connects two opposing sensing points. However, due to the rigid bonding at the $m_{n}$ points, the mechanical deformation of one segment (e.g., ${\mathrm{FS}}_{1}$) is decoupled from the deformation of the corresponding return segment (${\mathrm{FS}}_{3}$). It is this mechanical independence, despite the optical continuity, that is leveraged for the differential measurement scheme.

**Figure 3:**
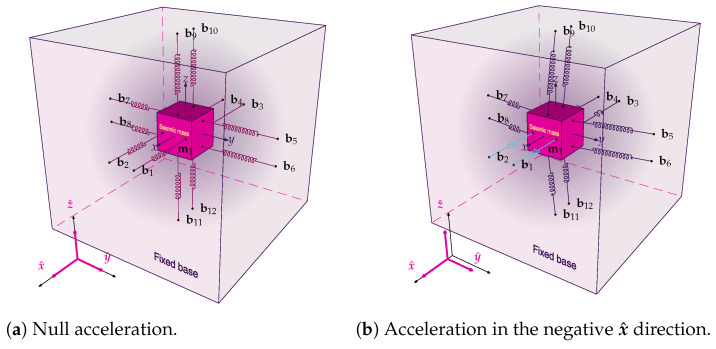

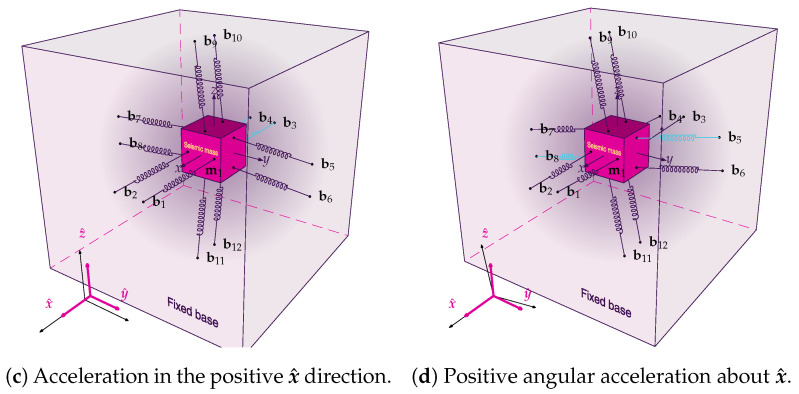
Connections of the seismic mass to the sensor base and its behavior under acceleration connections of the seismic mass to the sensor base and its behavior under acceleration. The FSs are numbered from ${\mathrm{FS}}_{1}$ to ${\mathrm{FS}}_{12}$, corresponding to the attachment points $b_{1}$ to $b_{12}$ on the base and $m_{1}$ to $m_{12}$ on the seismic mass, respectively. The ${\mathrm{FS}}_{1–4}$ are oriented along the $\hat{x}$ axis, the ${\mathrm{FS}}_{5–8}$ along the $\hat{y}$ axis, and the ${\mathrm{FS}}_{9–12}$ along the $\hat{z}$ axis.

Figure 3 illustrates how the acceleration causes deformation on OFs. The OFs are represented as springs, the colors cyan, purple and dark magenta, represent, respectively, compressed, tensioned and without deformation, when compared with tension on assembly. At rest (Figure 3a), the FSs maintain the assembly under tension (all FSs have the same deformation). An acceleration along the $-\hat{x}$ axis (Figure 3b) compresses FSs 1 and 2 and stretches FSs 3 and 4, while an acceleration along $+\hat{x}$ (Figure 3c) compresses FSs 3 and 4 and stretches FSs 1 and 2. A rotation around $+\hat{x}$ (Figure 3d) compresses FSs 5 and 8 and stretches FSs 6 and 7.

## 3. Methodology and Experimental Setup

The complete accelerometer system is composed of three main subsystems: the optomechanical assembly, an optical interrogation circuit, and an electronic data acquisition and processing circuit. Each of these subsystems is detailed below.

### 3.1. Optomechanical Design

The sensor employs a single seismic mass topology, consisting of an aerospace-grade aluminum cube with a side length of 16.3 mm  and a seismic mass of 11.61 g. This approach contrasts with typical modular designs, where each direction has a seismic mass with only one degree of freedom (DOF). The seismic mass is sustained by six optical fibers. Their fixing configuration forms twelve FSs, as shown in Figure 3, which act as spring elements. There is one FBG inscribed in each optical fiber, resulting in one FBG pair per axis. The pair is arranged in a push-pull configuration: one FBG is located on one side of the seismic mass, while the other is on the opposite side. Mechanically, accelerations induce a relative motion of the seismic mass with respect to the base, straining the fiber segments. Consequently, the FBGs measure this strain, which encodes the three-dimensional acceleration components. The design is inspired by the concept proposed by Cazo et al. [28].

The fiber segments were fixed to the sensor bases by applying a pre-tension of 2 N, using cyanoacrylate as the adhesive. This tension value corresponds to half the breaking strength of the OFs [38], and the tension was measured with a digital dynamometer. Fixation to the seismic mass was performed with epoxy resin, with a curing time of 18 h. In Figure 1, the sensor bases (in purple), also made of aluminum, represent the fixation points for the FSs (in blue), while the seismic mass is represented in pink. The sensor base is associated with the dynamics of the body whose acceleration is to be measured, whereas the seismic mass responds to both field accelerations (such as gravity) and accelerations originating from the base, which modify the tensions in the FSs.

### 3.2. Production and Characterization of FBG Sensors

**Figure 4:**
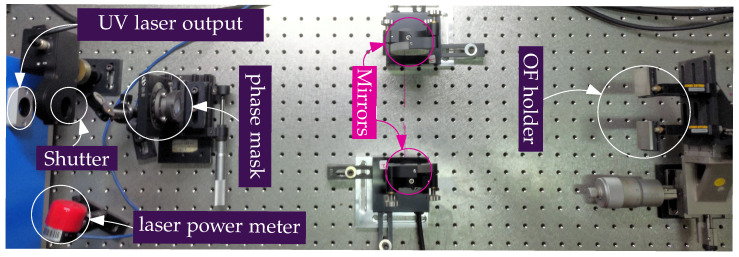
Experimental setup for the production of FBG sensors.

**Figure 5:**
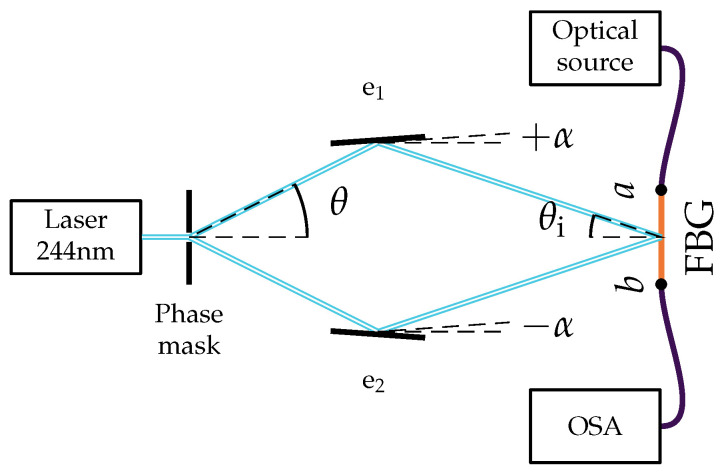
Schematic representation of the FBG production setup shown in Figure 4.

The FBG sensors were fabricated in-house to ensure control over their spectral characteristics. The fabrication process employed an interferometric setup based on the configuration described by [39]. A photograph of the setup is presented in Figure 4, and its schematic representation is shown in Figure 5. The diffraction half-angle (θ) of the first-order beams (m±1) generated by phase mask is determined by the mask period ($\Lambda_{\mathrm{pm}}$) and the wavelength of the inscription ultraviolet (UV) laser ($\lambda_{L}$), as described by [6]:

(3) $$\theta =\arcsin\frac{m\;\lambda_{L}}{\Lambda_{\mathrm{pm}}}.$$

The experimental setup comprises a UV laser ($\lambda_{L}=244\mathrm{nm}$, with an output power of 100 mW), a phase mask ($\Lambda_{\mathrm{pm}}=1052.6\;\mathrm{nm}$), two directional mirrors, an OF holder, and a shutter for exposure time control. Substituting the experimental parameters into Equation (3), the diffraction half-angle is calculated as:

(4) $$\theta =\arcsin\frac{1\;\cdot \;244\;\times \;10^{-9}}{1052.6\;\times \;10^{-9}}\approx 13.4{}^{\circ}.$$

Subsequently, the diffracted beams are redirected by the mirrors to interfere at the fiber core. The angle of incidence onto the fiber is defined as the half-intersection angle $\theta_{i}$.

The inscription period of the FBG (Λ) with this interferometric setup is given by [6]:

(5) $$\Lambda =\frac{\lambda_{L}}{2\sin\left(\theta -2\alpha \right)}.$$

Additionally, the Bragg condition states that the reflected wavelength is determined by the effective refractive index ($n_{\mathrm{eff}}$) and the grating period (Λ) [6]:

(6) $$\lambda_{g}=2n_{\mathrm{eff}}\Lambda .$$

Combining Equations (4)–(6), the expression that relates the desired Bragg wavelength to the angular adjustment α of the mirrors is obtained:

(7) $$\lambda_{g}=\frac{n_{\mathrm{eff}}\lambda_{L}}{\sin\left(\theta -2\alpha \right)}.$$

To inscribe an FBG with a desired Bragg wavelength, the angular deflection of the mirror can be calculated by solving Equation (7) for α:

(8) $$\alpha =\frac{\theta -\arcsin\frac{n_{\mathrm{eff}}\lambda_{L}}{\lambda_{g}}}{2}.$$

For example, to inscribe an FBG with a Bragg wavelength of 1.55 nm, in an OF with an effective refractive index estimated as 1.45, the angular deflection of the mirrors must be approximately:

(9) $$\alpha =\frac{13.4{}^{\circ}-\arcsin\frac{1.45\;\cdot \;244\;\mathrm{nm}}{1550\;\mathrm{nm}}}{2}\approx 0.10{}^{\circ}.$$

In practice, the procedure to determine the effective refractive index of an OF involves inscribing a preliminary FBG by setting the angle α to a known value. Then, the resulting Bragg wavelength is measured to obtain the effective refractive index using Equation (7). In this way, a more precise relationship between the angle α and the Bragg wavelength can be obtained with Equation (8) and the measured effective refractive index.

A germanium and boron co-doped optical fiber (Fibercore PS1250/1500), previously hydrogenated to increase its photosensitivity, was used for the grating inscription. A fundamental stage in the fabrication process involved real-time spectral characterization to monitor the grating reflectivity. This parameter is defined by the ratio between the reflected and incident optical power, associated with points *b* and *a* in Figure 5, respectively. The interrogation setup comprised an SLD source and an OSA (Advantest, model Q8347), configured with a spectral resolution of 7 pm, for signal acquisition.

The spectra acquired by the OSA are denoted by the vector p, with a dimension corresponding to the number of sampled wavelengths λ. Prior to UV exposure, a reference power spectrum, p(t=0) (in dBm), was recorded. During the inscription process, transmitted spectra p(t) (in dBm) were collected sequentially. Throughout the acquisition, the OSA maintained a constant wavelength range to ensure that reflectivity calculations were performed point-by-point according to Equation (11). The real-time reflectivity, r(λ,t), is determined by the inverse of the logarithmic relationship expressed in Equation (10).

(10) $$p\left(t=0\right)-p\left(t\right)=10\log_{10}\left(\frac{1}{1-r\left(\lambda \right)}\right).$$

(11) $$r\left(\lambda \right)=1-10^{\frac{p\left(t\right)-p\left(t=0\right)}{10}}.$$

**Figure 6:**
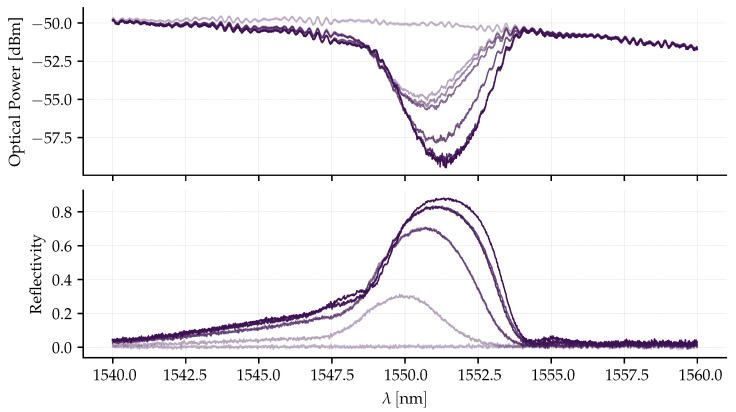
Data acquired during the production of an FBG with transmission interrogation. The ripples observed in the Optical Power graphic are static spectral artifacts of the inscription setup. As shown in the Reflectivity graph, these artifacts are eliminated by the differential calculatin described in Equation (11).

This monitoring, exemplified in Figure 6, allowed for the selection of FBGs with suitable reflectivity and spectral shape, preventing the sensor’s signal-to-noise ratio from being compromised. In the figure, the darker curves represent the most recent measurements, while the lighter ones correspond to the oldest, according to the inscription time. By monitoring the reflectivity, it is possible to prevent the development of side lobes in the FBG spectrum. With the presented setup, reflectivities greater than 80% were achieved with an exposure time of approximately 45 s.

**Figure 7:**
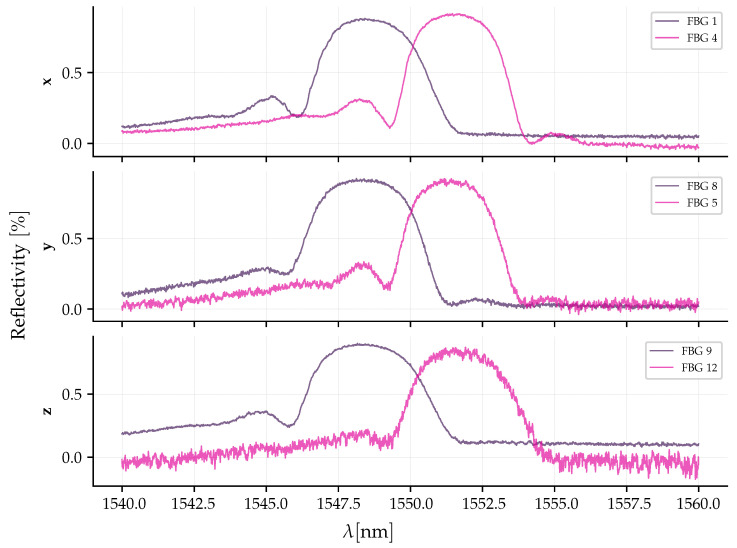
The FBG labels in the graphics correspond to the respective FSs illustrated in Figure 3. For instance, the curve designated FBG 1 corresponds to the specific grating inscribed on the fiber segment ${\mathrm{FS}}_{1}$, which is defined by points $b_{1}$ and $m_{1}$.

The three resulting pairs of FBGs, with slightly shifted reflectivity peaks to allow for differential measurement, are shown in Figure 7. The spectral bandwidth of the fabricated FBGs (≈3 nm) was specifically selected to optimize the sensor’s dynamic range. While narrower gratings would provide a steeper spectral slope (higher optical sensitivity), they would significantly restrict the linear measurement range, leading to early saturation. The chosen bandwidth ensures a monotonic response over the target range of $\pm 20\;g_{E}$, with the trade-off in optical sensitivity being compensated by the gain of the electronic signal conditioning stage. Furthermore, the objective was to design the pairs so that, under zero acceleration, the crossing point between their spectra occurs in the region of steepest slope, corresponding to 50% of the maximum reflectivity. This configuration maximizes the effective full-scale range from an optical perspective, as will be discussed in Section 3.4.

### 3.3. Differential Optical Interrogation Circuit

**Figure 8:**
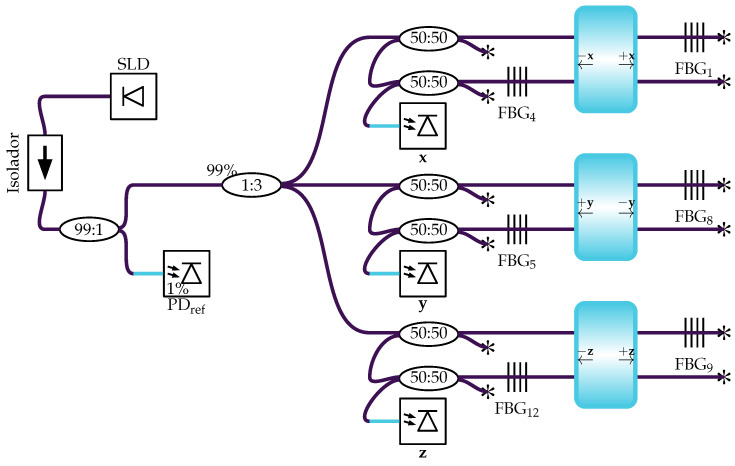
Diagram of the differential optical circuit for the three accelerometer axes. The asterisk (∗) indicates that impedance matching was implemented to eliminate light return due to reflection at the fiber optic tip. The blue block represents a schematic cut-view of the seismic mass where the arrows indicate the connection points and sensing directions of the FBG pairs. For example, on the *x* axis, the ${\mathrm{FBG}}_{4}$ connection is associated with the segment oriented in the $+\hat{x}$ direction, and ${\mathrm{FBG}}_{1}$ with the $-\hat{x}$ direction.

The operating principle of the accelerometer is based on the differential interrogation technique, implemented through a dual-reflection topology. The complete optical circuit, illustrated in Figure 8, integrates the stabilized optical source with the three measurement channels, corresponding to the sensitive axes *x*, *y*, and *z*. The optical source is composed of an SLD (DenseLight DL-CS5203A), whose stability is ensured by an optical isolator. The emitted light is sequentially divided: a 99:1 coupler splits a fraction of the power to a reference photodetector (${\mathrm{PD}}_{\mathrm{ref}}$) in the 1% channel to mitigate the effects of source fluctuations, while the main channel (99%) is subsequently split by a 1:3 coupler, distributing the power to the three distinct outputs (x, y, and z) which serve as sources for the interrogation circuits of each axis. Each axis utilizes an identical differential interrogation circuit composed of a 50:50 coupler and a pair of FBGs that act as differential sensors. This configuration physically implements the differential measurement method, whose conceptual basis assumes that the acceleration along an axis can be inferred directly from the strain difference between a pair of opposing FBGs. The FBG pairs are arranged in a “push-pull” configuration, where an acceleration along the sensitivity axis induces tension in one grating and simultaneous compression in the other, resulting in spectral shifts in opposite directions. The differential measurement of the separation between the Bragg peaks doubles the system’s sensitivity compared to a single-grating sensor and promotes the rejection of common-mode disturbances, such as temperature fluctuations [28]. The FBG labels in the graphics correspond to the respective FSs illustrated in the accelerometer structure (Figure 3), where the term FBG denotes the Bragg grating inscribed within the fiber core. For instance, ${\mathrm{FBG}}_{1}$ and ${\mathrm{FBG}}_{4}$ are aligned with the *x*-axis, ${\mathrm{FBG}}_{8}$ and ${\mathrm{FBG}}_{5}$ with the *y*-axis, and ${\mathrm{FBG}}_{9}$ and ${\mathrm{FBG}}_{12}$ with the *z*-axis, mapping directly to the specific fiber segments and connection points (e.g., ${\mathrm{FBG}}_{1}$ is between $b_{1}$ and $m_{1}$) shown in the three-dimensional diagram (Figure 3). All optical components used in the sensor, including OFs, couplers, and the isolator, are of the single mode (SM) type.

The reflectivity spectra of the utilized FBGs have a bandwidth of approximately 3 nm, whereas broadband sources like SLDs have spectra of approximately 40 nm. To analyze the interrogation equations, it is assumed that, in the operating region, the spectrum of the broadband source is approximately flat and can be modeled by:

(12) $$\begin{array}{cc} s\left(\lambda \right)=\; & A_{b}, \end{array}$$

where $A_{b}$ [W/m] represents the amplitude of the broadband source spectrum. The optical power read by the photodetector in the dual-reflection interrogation, shown in Figure 8, is modeled by:

(13) $$\begin{array}{cc} p^{\mathrm{pd}}\left(\lambda \right)=\; & \frac{A_{b}}{16}\int r_{2}\left(\lambda \right)r_{1}\left(\lambda \right)d\lambda . \end{array}$$

The terms $r_{1}\left(\lambda \right)$ and $r_{2}\left(\lambda \right)$ represent the reflectivity spectrum of the pairs for one axis as a function of wavelength λ. The total power incident on the photodetector is obtained by integrating Equation (13) over the entire spectrum. The factor of 16 results from four passes of the light through the couplers.

The choice of this differential intensity-based interrogation architecture, as opposed to standard wavelength division multiplex (WDM) approaches, is strictly driven by the requirements of embedded aerospace applications. While WDM offers superior multiplexing density, it typically necessitates complex spectral analysis hardware—such as tunable lasers or high-resolution spectrometers—to track individual Bragg wavelengths. These devices are generally bulky, expensive, and power-intensive, making them less suitable for small-satellite or sounding rocket payloads with severe SWaP constraints.

In contrast, the proposed topology converts the wavelength shift directly into an optical power variation through the spectral overlap of the FBG pairs. This allows the demodulation to be performed by a fully passive optical circuit coupled with simple photodetectors and standard operational amplifiers (as detailed in Section 3.5). This architecture not only reduces the system’s footprint and cost but also provides a high-bandwidth analog output suitable for real-time control loops, avoiding the sampling rate limitations often found in low-cost scanning interrogators. Furthermore, this architecture offers the potential for future integration into a complete optical inertial measurement unit (IMU), where the broadband light source could be shared with a interferometric fiber-optic gyroscope (IFOG), significantly optimizing the system’s overall power budget and footprint.

### 3.4. Full-Scale Range Analysis

The determination of the accelerometer’s full-scale range was carried out from two perspectives: the mechanical limit, related to the structural integrity of the fibers, and the optical limit, imposed by the nature of the interrogation system. The fiber segment is modeled as a linear spring with stiffness k, as described by [40]. It is important to note that acrylate coating was mechanically stripped in the sensing region (the FS between $m_{m}$ and $b_{m}$) prior assembly. This eliminates viscoelastic creep effects and ensures that the stiffness is governed solely by the silica cladding proprieties. Assuming a Young’s modulus for silica of Y=70 GPa, an optical fiber diameter of ⌀=125 μm, and an initial length of $\ell_{0}=3\mathrm{nm}$, the stiffness k was calculated as:

(14) $$k=\frac{Y\frac{\pi {⌀}^{2}}{4}}{\ell_{0}},$$

resulting in k≈286.34 kN/m. From this value, the initial strain $\varepsilon_{a}$, due to the pre-tension $T_{a}$ of 2 N, was determined as:

(15) $$\begin{array}{cc} \varepsilon_{a}=\frac{\Delta \ell_{0}}{\ell_{0}} & =\frac{T_{a}}{k\ell_{0}}=0.0023. \end{array}$$

For the estimation of the accelerometer’s full-scale range, the linearized dynamics relationship was considered:

(16) $$m_{m}\ddot{r}=4k\Delta \ell_{0},$$

in which $m_{m}$ and $\ddot{r}$ represent the seismic mass and acceleration, respectively, and $\Delta \ell_{0}$ is the change in length of the fiber segment. The factor 4k arises from the parallel combination of the fiber segments. By substituting Equation (14) into Equation (16), the measured strain $\varepsilon_{m}$ can be expressed as a function of acceleration, yielding:

(17) $$\begin{array}{cc} \varepsilon_{m} & =\frac{m_{m}\ddot{r}}{Y\pi {⌀}^{2}}. \end{array}$$

The two strain components, $\varepsilon_{a}$ and $\varepsilon_{m}$, which cause the spectral shifts, make up the total strain imposed on each fiber segment. Thus, the Bragg equation (Equation (1)) can be rewritten to provide the relationship between the change in Bragg wavelength $\Delta \lambda_{g}$ and the strains $\varepsilon_{m}$ and $\varepsilon_{a}$:

(18) $$\frac{\Delta \lambda_{g}}{\lambda_{g}}=\left(1-p_{e}\right)\left(\varepsilon_{m}+\varepsilon_{a}\right)+\left(\alpha +\zeta \right)\Delta T.$$

It is observed that the measured strain $\varepsilon_{m}$ can be positive or negative, depending on the position relative to the seismic mass and the direction of acceleration. The breaking strain of the PS1250/1500 optical fiber (without the protective jacket) is 0.006 [38]. Therefore, the accelerometer must operate within the strain range:

(19) $$0<\left|\varepsilon_{a}+\varepsilon_{m}\right|<0.006.$$

The inequality in Equation (19) shows that the total strain $\varepsilon_{a}+\varepsilon_{m}$ must remain below the OFs breaking point and above zero strain to maintain structural integrity. In the case of compression, where $\varepsilon_{m}$ is negative, to avoid fiber slackness (loss of traction), it is necessary that $\varepsilon_{m}>-\varepsilon_{a}$; therefore, based on Equation (15), $\varepsilon_{m}>-0.0023$. Conversely, under traction traction, the limit in Equation (19) dictates that $\varepsilon_{m}<0.006-\varepsilon_{a}$, resulting in $\varepsilon_{m}<0.0037$. Since the symmetric operational ranges is constrained by tighter of these two limits, it is assumed $\left|\varepsilon_{m}\right|<\varepsilon_{a}$. Substituting this into to Equation (17), the maximum allowable acceleration is:

(20) $$\left|{\ddot{r}}_{max}\right|<\frac{\varepsilon_{a}Y\pi {⌀}^{2}}{m_{m}}=672.00\;m/s^{2}\left(68.50\;g_{E}\right).$$

Based on the sensor topology, where the seismic mass of $m_{m}=16.3\;g$ is suspended solely by the four optical fiber segments acting as elastic elements, the theoretical sensitivity, 𝒮, can be estimated analytically. By substituting the stiffness and deformation definitions from Equations (2) and (16) into the Bragg relation Equation (1), the sensitivity—defined as the ratio between the Bragg wavelength shift $\Delta \lambda_{g}$ and the applied acceleration $g_{E}$—is given by:

(21) $$𝒮=\frac{\lambda_{g}(1-p_{e})m_{m}}{4k\ell_{0}}.$$

Considering the spring of a single fiber segment k on Equation (14), the photo-elastic coefficient of silica $p_{e}\approx 0.21$, the initial length of the FS $\ell_{0}=3\;\mathrm{nm}$, and a center wavelength of 1550 nm, the predicted spectral sensitivity for a single FBG is approximately $\approx 56\;pm/g_{E}$. Due to the differential interrogation scheme employed, the effective sensitivity is doubled, resulting in a theoretical sensor sensitivity of ≈$112\;pm/g_{E}$ for each axis.

Furthermore, the mechanical bandwidth is governed by the natural frequency of the mass-spring system, defined as fn=12π4kmm. For the implemented design parameters, the theoretical resonant frequency is approximately 1.33 kHz. Following standard inertial sensor design principles, the usable flat bandwidth is typically estimated as one-third of the resonant frequency to minimize amplitude distortion. Thus, the proposed sensor offers an estimated operational bandwidth of approximately 440 Hz, which comfortably covers the bandwidth required for tactical navigation (typically <100 Hz). Although the dynamic response was not characterized in a vibration table due to mission integration constraints, this theoretical value aligns with the operational requirements of the interrogation system.

However, beyond the structural limits, the sensor’s operating range is fundamentally restricted by the differential optical interrogation system. In this dual-reflection scheme, the detected optical power is governed by the spectral overlap between the reflections peaks of a pair of FBGs. As shown in Figure 7, the initial spectral separation between the peaks of the FBGs pairs at rest is approximately $\Delta \lambda_{g,sep}\approx 2.5\;nm$.

Given the differential sensitivity of the sensor, estimated at ≈$112\;pm/g_{E}$, the theoretical acceleration required to cause the spectral to crossing, leading to a loss of injectivity, can be calculated as:

(22) $${\ddot{r}}_{limit}\approx \frac{\Delta \lambda_{g,sep}}{𝒮}\to \frac{2500\;pm}{112\;pm/g_{E}}\approx 22.3g_{E}.$$

**Figure 9:**
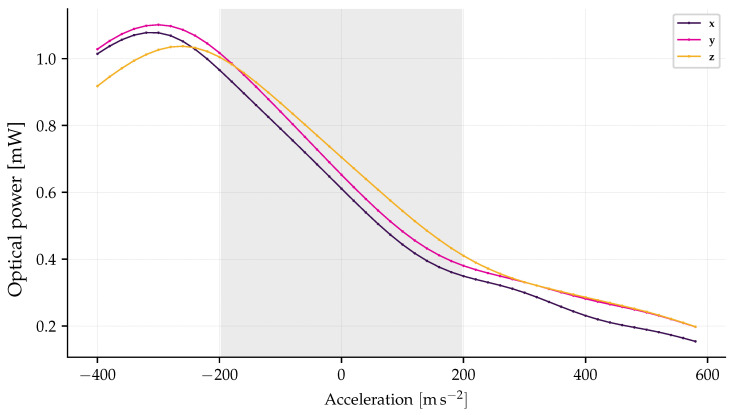
Relationship between optical power and acceleration.

Beyond the range of $\approx \pm 22\;g_{E}$, the function relating acceleration to optical power becomes non-injective, as confirmed by the numerical simulation presented in Figure 9. Consequently, to ensure a safe margin against measurement ambiguity and saturation, the effective full-scale range was defined as $\pm 20\;g_{E}$.

To evaluate the linearity performance within this theoretically defined range, a numerical simulation was performed using the experimental spectral profiles of the fabricated FBGs, based on the theoretical model in Equation (13). The results, presented in Figure 9, illustrate the convolution response behavior. The greyed regions in the figure highlight the defined operational range of $\left[-20\;g_{E},\;20\;g_{E}\right]$. Within this interval, the simulation confirms that the sensor maintains a monotonic and injective mapping, guaranteeing that each power measurement corresponds to a unique acceleration value. Outside this range (e.g., below approximately $-22\;g_{E}$), the relationship becomes non-injective due to the spectral crossing phenomena described above. Therefore, the simulation serves to validate the linearity of the response inside the limits imposed by the spectral separation.

To quantify the adherence to a linear model, a non-linearity error analysis was performed over this full-scale range. For each axis, a linear regression was applied to the convolution results to determine the optimal slope and intercept coefficients. The non-linearity error $\epsilon_{nl}$ is defined as the maximum deviation between the simulated optical power and the linear fit, normalized by the full-scale (FS) output:

(23) $$\epsilon_{nl}=\frac{\max(|y_{m}-y_{f}|)}{f_{s}}\times 100,$$

where $y_{m}$ represents the data points derived from the convolution and $y_{f}$ the corresponding values from the linear regression. This metric provides a standardized assessment of the sensor’s proportional accuracy across its operating dynamic range.

### 3.5. Signal Conditioning and Data Acquisition Electronics

Due to the low optical power levels resulting from the differential interrogation (on the order of microwatts), a transimpedance amplifier circuit is used to convert the generated photocurrent into a measurable voltage signal. The circuit employs a low-noise operational amplifier (AMP OP) (OPA2727) and a photodetector with a responsivity of $k^{\mathrm{pd}}\approx 0.9\;A/W$. A first-order low-pass filter, formed by a capacitor in parallel with the feedback resistor, is implemented to limit the bandwidth and attenuate high-frequency noise.

Digital processing and data acquisition are performed by an ESP32 microcontroller, which integrates two 12-bit analogic-to-digital converters (ADC). The raw ADC count values for each axis are normalized by the count from the power reference channel to compensate for source fluctuations, generating the normalized measurement vector $\bar{\Psi_{m,i}}$ for the *i*-th sample, according to:

(24) $$\bar{\Psi_{m,i}}=\frac{1}{c_{\mathrm{ref},i}^{\mathrm{tr}}}\left[\begin{array}{c} c_{x,i}^{\mathrm{tr}}\\ c_{y,i}^{\mathrm{tr}}\\ c_{z,i}^{\mathrm{tr}} \end{array}\right],$$

where $c_{\mathrm{ref},i}^{\mathrm{tr}}$ is the count from the reference channel, and $c_{x,i}^{\mathrm{tr}}$, $c_{y,i}^{\mathrm{tr}}$, and $c_{z,i}^{\mathrm{tr}}$ are the counts from each axis.

The value of the feedback resistor $R_{l}^{\mathrm{tr}}$ for the *ı*-th axis can be sized to obtain an output voltage of $v^{\mathrm{tr}}=1.5\;V$ for a given incident optical power level $p_{l}^{\mathrm{pd}}$, and is given by $R_{l}^{\mathrm{tr}}=p_{l}^{\mathrm{pd}}\;\cdot \;v^{\mathrm{tr}}\;/\;\;k^{\mathrm{pd}}$. Meanwhile, the capacitor is determined by ${C^{\mathrm{tr}}}_{l}={\left(2\pi \;\cdot \;f^{\mathrm{tr}}\;\cdot \;R_{l}^{\mathrm{tr}}\right)}^{-1}.$

For the sensor’s benchtop characterization, particularly the bias instability analysis via Allan Deviation, a data acquisition setup was implemented. This system combined an analog low-pass filter, with a cutoff frequency ($f^{\mathrm{tr}}$) designed at ≈1 Hz, and a digital sampling rate ($f^{s}$) of 100Hz.

This configuration is ideal for static noise characterization. The analog filter serves as a robust anti-aliasing filter, maximizing the attenuation of high-frequency noise. This is particularly effective at rejecting specific disturbances common in a laboratory environment, such as low-frequency building microvibrations and thermal chamber compressor noise. Concurrently, the 100 Hz sampling rate provides significant oversampling of the signal band of interest ($f^{s}\gg 2\;f^{\mathrm{tr}}$).

This combination ensures the quasi-static signal is digitized with high fidelity. While this filtering attenuates high-frequency white noise, thereby improving the measured VRW relative to a broader bandwidth dynamic application, it does not distort the bias instability measurement. Since the bias instability phenomenon dominates at long correlation times ($\tau \gg 1/f_{c}$), it remains unaffected by the 1 Hz low-pass cutoff, preserving the validity of this critical tactical-grade metric.

It must be emphasized, however, that this cutoff frequency is specific to the static testing protocol. For the final application in dynamic navigation, the filter must be redesigned for a higher cutoff frequency (e.g., 50 Hz). This is necessary to avoid introducing significant phase lag into the signals of interest (aircraft rigid-body modes), which would otherwise compromise the inertial integration process.

For the thermal compensation routine, the system temperature is monitored by a digital sensor (Model AHT10, Aosong Electronics), featuring an accuracy of ±0.3 °C and resolution of 0.01 °C. This sensor is integrated into the acquisition electronics located inside the sealed sensor housing. Since the unit is factory-calibrated and used consistently for both the calibration procedure and operational flight, any absolute bias in the temperature reading is effectively absorbed by the polynomial model’s bias coefficients, $b_{cT(k)}$ (detailed in Section 3.6.2). Furthermore, the sensor PCB is mechanically anchored to the accelerometer’s aluminum structure. This arrangement ensures that the measured temperature is representative of the optomechanical assembly’s thermal equilibrium, thereby minimizing the thermal lag between the structural expansion and the compensation variable.

The development of this custom interrogation system, as opposed to using commercial off-the-shelf (COTS) interrogators, is justified by the specific requirements of the intended aerospace application. First, commercial WDM interrogators based on spectral scanning often exhibit limited sampling rates (typically <1 kHz for compact units). In the vibration-rich environment of a launch vehicle, this limitation precludes the detection of high-frequency structural modes and makes the navigation data susceptible to aliasing. In contrast, the proposed custom electronics provide a continuous high-bandwidth analog output, allowing for effective anti-aliasing filtering and dynamic analysis limited only by the cutoff frequency of the transimpedance amplifier.

Furthermore, the system is designed as a fully integrated optoelectronic architecture rather than a generic sensor-reader pair. The “interrogator” functionality is intrinsic to the optical domain, relying on the specific spectral overlap between the sensor and reference FBGs to perform the signal demodulation. This dedicated design philosophy allows for a drastic reduction in SWaP and cost compared to general-purpose spectral analyzers, making the sensor suitable for embedding in sounding rockets and microsatellite platforms where volume and energy budget are critical constraints.

### 3.6. Characterization Procedures and Calibration Model

The characterization of the accelerometer involved multiple procedures to evaluate its static, dynamic, and thermal performance. Static calibration was performed using the reference field method. The sensor was mounted on a two-degree-of-freedom rotation table, allowing it to be positioned in various known orientations relative to the Earth’s gravitational field. From the normalized sensor readings and the reference acceleration vectors, the 12 parameters of a linear calibration model (compensation matrix $M_{\Psi }$ and bias vector $b_{c}$), according to Equation (25), were estimated via least squares method (LSQ).

(25) $$\Psi_{c,i}=M_{\Psi }\bar{\Psi_{m,i}}+b_{c}.$$

This calibration method is described in detail in the works of [34,35]. With the parameters obtained in this step, while maintaining a constant temperature, it was possible to perform the bias instability test, presented in the next section. In Section 3.6.2, a modification of this method is presented to include thermal compensation.

#### 3.6.1. Bias Instability and Velocity Random Walk

The inherent noise in an accelerometer’s measurements, when integrated to obtain velocity, results in an error that grows over time. This characteristic is quantified by the VRW parameter, denoted as $\sigma_{VRW}$. Another crucial parameter for characterizing the long-term performance of the sensor is the bias instability ($\sigma_{b}$), which describes the slow fluctuations or drift of the mean bias value over time. To quantitatively estimate the $\sigma_{VRW}$ and $\sigma_{b}$ parameters, the Allan variance (AVAR) method was adopted.

The value of $\sigma_{VRW}$ is obtained from the Allan Deviation ($\sigma_{a}$, which is the square root of the Allan Variance, computed using the AllanTools Python library [41]) for a correlation time τ of 1 s, given by:

(26) $$\begin{array}{cc} \sigma_{VRW}=\; & 60\;\cdot \;\sigma_{a}\left(\tau =1s\right)\left[m/s/\sqrt{h}\right]. \end{array}$$

while the $\sigma_{b}$ is associated with the minimum value of the Allan Deviation curve [36].

(27) $$\sigma_{b}=\frac{{\left.\sigma_{a}\right|}_{\frac{\partial \sigma }{\partial \tau }=0}\;\times \;10^{3}}{0.66\;\times \;9.81}\left[mg_{E}\right].$$

**Figure 10:**
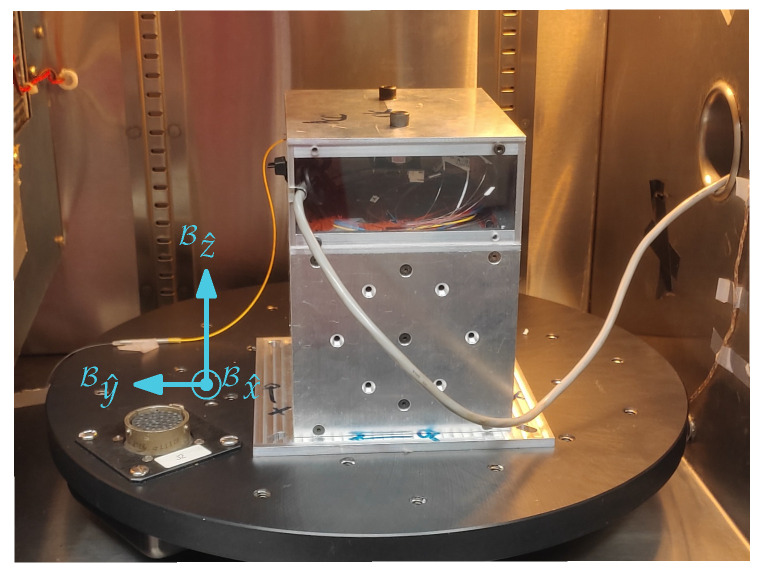
Accelerometer inside a thermal chamber for a static temperature test without attitude variation.

For the bias instability assessment, the sensor was kept at rest inside a thermal chamber (Figure 10) at a controlled temperature of 23.4 °C. The data were transmitted via Bluetooth and stored on a computer for post-processing. For the Allan variance analysis, the last six hours of acquisition were considered to ensure the sensor was in thermal equilibrium.

#### 3.6.2. Calibration Model with Thermal Compensation

The linear model (Equation (25)), although straightforward to implement, does not account for temperature effects. Although the differential topology has the potential to mitigate common-mode thermal disturbances in the sensing element, the overall system performance remains susceptible to temperature variations in peripheral optical components. The response of passive components, such as optical couplers, exhibits thermal dependence, with variations in insertion loss and coupling ratio [31,32]. Additionally, optical sources like the SLD show power and wavelength variations with temperature [33]. These systemic instabilities can manifest as bias and scale factor errors, reinforcing the need for a calibration model that actively compensates for the thermal dependence of the entire measurement chain.

To compensate for these errors, a polynomial calibration model with degree *n* is proposed, which extends the linear model by incorporating temperature-dependent terms. The relationship between the reference measurement ($\Psi_{c}$) and the normalized raw measurement ($\bar{\Psi_{m}}$) is modeled as:

(28) $$\Psi_{c}=\sum_{k=0}^{n}\left(M_{\Psi T(k)}T^{k}\right)\bar{\Psi_{m}}+\sum_{k=0}^{n}\left(b_{cT(k)}T^{k}\right).$$

The choice of a polynomial structure of order *n* (where n>1) is physically justified by the aggregate behavior of the optoelectronic chain. While the thermal response of the FBG sensor itself is dominated by the linear thermo-optic coefficient of silica α (≈$8.6\;\times \;10^{-6}/{}^{\circ}C$) over the tested range, the interrogation system introduces significant non-linear dependencies. Specifically, the broadband SLD source exhibits a temperature-dependent wavelength drift and spectral shape variation (typically Gaussian). Since the measurement principle relies on the convolution of this source spectrum with the FBG reflection profiles (as detailed in Equation (13)), a linear shift in temperature results in a non-linear variation in the integrated optical power. Furthermore, the splitting ratio of the optical couplers and the responsivity of the photodetectors also exhibit thermal sensitivities. Consequently, the polynomial coefficients $M_{\Psi T(k)}T^{k}$ and $b_{cT(k)}T^{k}$ for k≥2 in Equation (28) act as lumped parameters to compensate for these combined systemic non-linearities, which cannot be corrected by a simple bias and scale factor adjustment.

The fundamental difference between the calibration model defined in Equation (28) and the linear approach of Equation (25) lies in the explicit inclusion of thermal effects. Temperature dependence is incorporated into the scale factor matrix through the term $M_{\Psi T(k)}T^{k}$, as well as into the bias vector via $b_{cT(k)}T^{k}$.

The model has a total of $N_{p}=12\left(n+1\right)$ scalar parameters to be estimated. For this, Equation (28) is rewritten as a linear matrix system in the form G=AX. For a set of *m* measurements collected at different orientations and temperatures, the matrices are defined as follows:

- $G\in {\mathbb{R}}^{3\times m}$ is the matrix of reference accelerations, where each column corresponds to the vector $g_{i}$ for measurement *i*.
- $A\in {\mathbb{R}}^{3\times 4(n+1)}$ is the parameter matrix to be estimated, containing the coefficients $M_{\Psi T(k)}$ and $b_{cT(k)}$.
- $X\in {\mathbb{R}}^{4(n+1)\times m}$ is the regressor matrix. Each column $X_{i}$ is constructed from the raw measurement $\bar{\Psi_{m,i}}$ and the temperature $T_{i}$, with the following structure:
  (29) $$X_{i}={\left[{\bar{\Psi_{m,i}}}^{⊤},\;{\left(\bar{\Psi_{m,i}}T_{i}\right)}^{⊤},\;\ldots ,\;{\left(\bar{\Psi_{m,i}}T_{i}^{n}\right)}^{⊤},\;1,\;T_{i},\;\ldots ,\;T_{i}^{n}\right]}^{⊤}.$$

The parameter matrix A that minimizes the quadratic error, $||G-{AX||}_{F}^{2}$, is found by the LSQ solution, given by:

(30) $$\hat{A}=GX^{⊤}{\left(XX^{⊤}\right)}^{-1}.$$

The expression $||G-{AX||}_{F}^{2}$ represents the squared Frobenius norm of the residual error matrix. The resulting difference matrix, E=G−AX, aggregates the estimation errors for all *m* measurements and for the three axes. The Frobenius norm is defined as the square root of the sum of the squares of all matrix elements, analogous to the Euclidean norm for vectors. Therefore, minimizing the square of this norm is precisely the objective of LSQ: to find the parameter matrix $\hat{A}$ that minimizes the total sum of squared errors across the entire dataset.

Conceptually, this estimation problem is equivalent to finding the affine transformation that maps the distorted ellipsoid, formed by the raw measurements at different orientations, back to the ideal sphere with a radius of $1\;g_{E}$, since gravity is used as the reference in this case. The scale factor matrix corrects the ellipsoid’s distortion, and the bias ensures that the ellipsoid is centered at the origin.

Although geometric fitting methods exist [42], the algebraic LSQ approach was adopted here for two strategic reasons. First, its computational simplicity allows for an efficient implementation on embedded hardware [35]. Second, the algebraic formalism permits a direct extension of the model to incorporate thermal dependencies, providing the foundation for the proposed comprehensive calibration model. The choice of the polynomial degree, *n*, represents a trade-off between model fidelity and the risk of overfitting.

**Figure 11:**
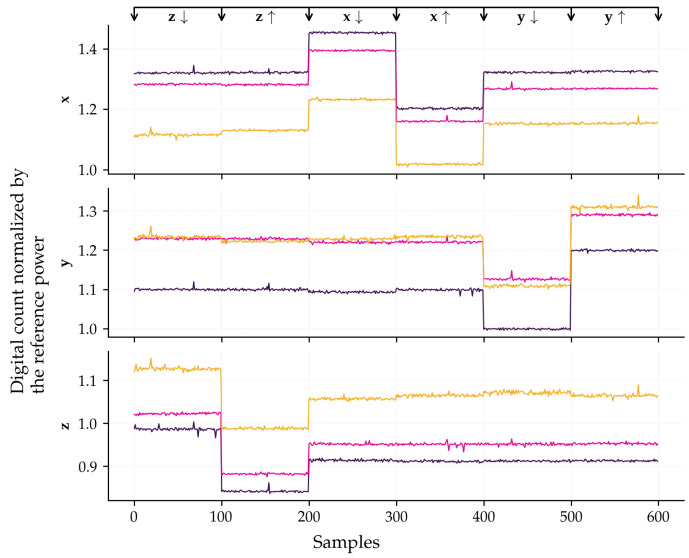
Data acquired for calibration under controlled temperature conditions. The colors purple, magenta, and yellow represent 16 °C, 24 °C, and 45 °C, respectively. At the top of the graph, the axis that is parallel to the gravity vector is indicated, where the notation z↓ signifies that the respective axis (z) is oriented downward. In this setup, the other two axes are orthogonal to gravity and, ideally, should exhibit zero measurement. However, the acquired data reveals significant cross-axis coupling effects. The subsequent section presenting the results demonstrates the capability of the proposed calibration method to effectively correct these parasitic effects.

For the thermal characterization, the calibration procedure was repeated at different controlled temperatures (16  °C, 24 °C, and 45 °C) using a testbed with temperature control and three-degree-of-freedom rotation. Figure 11 presents the data acquired for calibration, already normalized by the reference measurement. The test is performed by pointing one axis at a time vertically downwards and upwards. The first axis was the z-axis, with a 180° rotation around the y-axis. Then, the x-axis was subjected to the same procedure, followed by the y-axis.

It is worth noting that, to ensure the metrological reliability required for such high-performance applications, the characterization of modern accelerometers increasingly follows standardized guidelines, such as the IEEE Std 1293-2018 [26]. However, while the aforementioned studies focus primarily on optimizing sensitivity and frequency response, a critical and often overlooked aspect is the rigorous characterization of thermal bias stability under standardized protocols. This focus is justified because the bias stability is the governing parameter during the initial alignment and ‘warm-up’ phases of inertial navigation systems, determining the accuracy of the attitude estimation while the vehicle is stationary.

## 4. Results and Discussion

This section presents the experimental results of the accelerometer characterization, including the validation of the conditioning circuit, static calibration, instability analysis, and the evaluation of the thermal compensation model.

### 4.1. Conditioning Circuit Characterization

The first experimental stage consisted of validating the signal conditioning circuit. Table 1 presents the optical power levels measured in each channel under zero acceleration conditions. Based on these values and the responsiveness of the photodetector, the resistors (*R*^tr^) and capacitors (*C*^tr^) of the transimpedance amplifier were sized, as discussed in Section 3.5. The commercial values used and the resulting cutoff frequency are also presented, demonstrating the circuit’s compliance with the design parameters.

### 4.2. Linearity Analysis via Spectral Convolution

**Figure 12:**
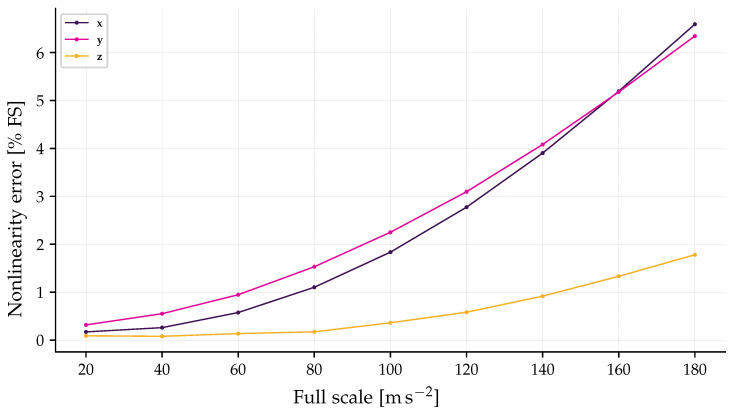
Non-linearity error as a function of the full-scale acceleration for the three orthogonal axes.

The linearity performance of the triaxial accelerometer was evaluated for the x, y, and z axes by analyzing the convolution of the FBGs spectra, as illustrated in Figure 12. This analysis, based on the relationship between optical power and acceleration shown in Figure 9, indicates that the non-linearity error remains below 6.7%  of the FS for all axes within the range up to 180 m/$s^{2}$.

In this characterization, the x and y axes exhibited the highest deviation, reaching approximately 6.6% and 6.3% at the upper limit of the scale, respectively. Conversely, the *z*-axis demonstrated superior linear stability, presenting the lowest non-linearity error. This behavior is attributed to the inherent spectral profiles of the inscribed gratings. As observed in Figure 7, the spectra of the six FBG segments are not identical; variations in bandwidth, reflectivity, and side-lobe suppression ratio (SLSR) result in distinct convolution integrals for each segment. Consequently, as the acceleration increases and shifts the Bragg wavelengths, the non-uniformity of the spectral shapes introduces slight non-linearities in the power-to-acceleration mapping. Despite these variations, the results remain predictable and suitable for compensation through the calibration model described in Section 3.6.

### 4.3. Static Calibration at Constant Temperature

**Figure 13:**
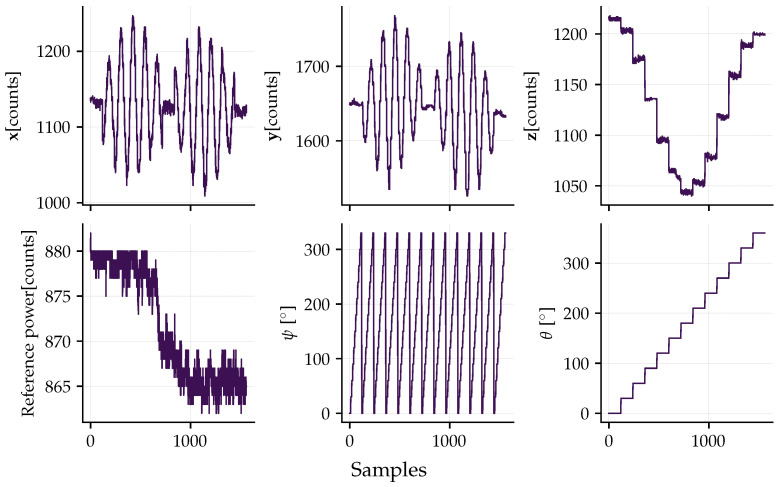
Raw data dataset acquired during the static calibration procedure performed at a constant temperature of ≈23.4 °C. The top row displays the uncalibrated response (in digital counts) for the $\hat{x}$, $\hat{y}$, and $\hat{z}$ axes as the sensor undergoes discrete rotations. The bottom row presents the auxiliary variables: the optical reference power (used for normalization), the rotation angle ψ (continuous rotation around the table axis), and the inclination angle θ (discrete steps relative to gravity).

The static calibration was performed at a temperature of 23.4 °C. Figure 13 illustrates the acquired raw dataset, the reference power, and the orientation to which the sensor was subjected. The test was conducted by rotating the sensor around the ψ axis and incrementing the θ angle after each full turn. In both cases, the increments were 30°. With these angles, the local gravity vector was transformed into accelerations in the sensor’s fixed frame and stored in the reference measurement matrix.

**Figure 14:**
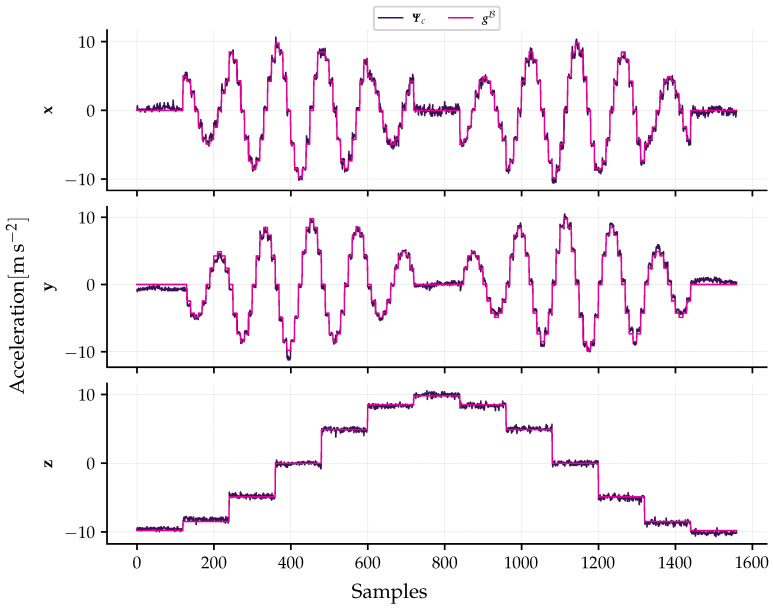
Validation of the static calibration at 23.4 °C. The plots compare the calibrated sensor acceleration ($\Psi_{c}$, purple line) against the gravitational reference field ($g^{B}$, magenta line) for the three orthogonal axes. The precise overlap between the measured and reference curves demonstrates the effectiveness of the estimated compensation matrix $M_{\Psi }$ and bias vector $b_{c}$ in correcting scale factor and misalignment errors.

The application of the reference-field calibration method to the input data and reference acceleration vectors resulted in the compensation matrix ($M_{\Psi }$) and the bias vector ($b_{c}$), whose values are presented in Equation (31a,b). The effectiveness of the calibration is evidenced in Figure 14, which displays the data after applying the model. It is observed that the compensated measurements of the three axes follow the gravitational field reference, validating the linear model for a fixed operating temperature.

It is worth noting that the measurements for each axis must be normalized by the reference measurement. Thus, the elements of the $M_{\Psi }$ matrix are dimensionless, while the terms of the $b_{c}$ vector are in units of acceleration, $m/s^{2}$.

(31a) $$\begin{array}{cc} M_{\Psi } & =\left[\begin{array}{ccc} -81.60 & 2.89 & -0.71\\ 1.59 & 77.66 & 3.75\\ -0.24 & 1.23 & -107.66 \end{array}\right], \end{array}$$

(31b) $$\begin{array}{cc} b_{c} & ={\left[\begin{array}{ccc} 101.08 & -153.59 & 137.16 \end{array}\right]}^{⊤}. \end{array}$$

### 4.4. Bias Instability Analysis

**Figure 15:**
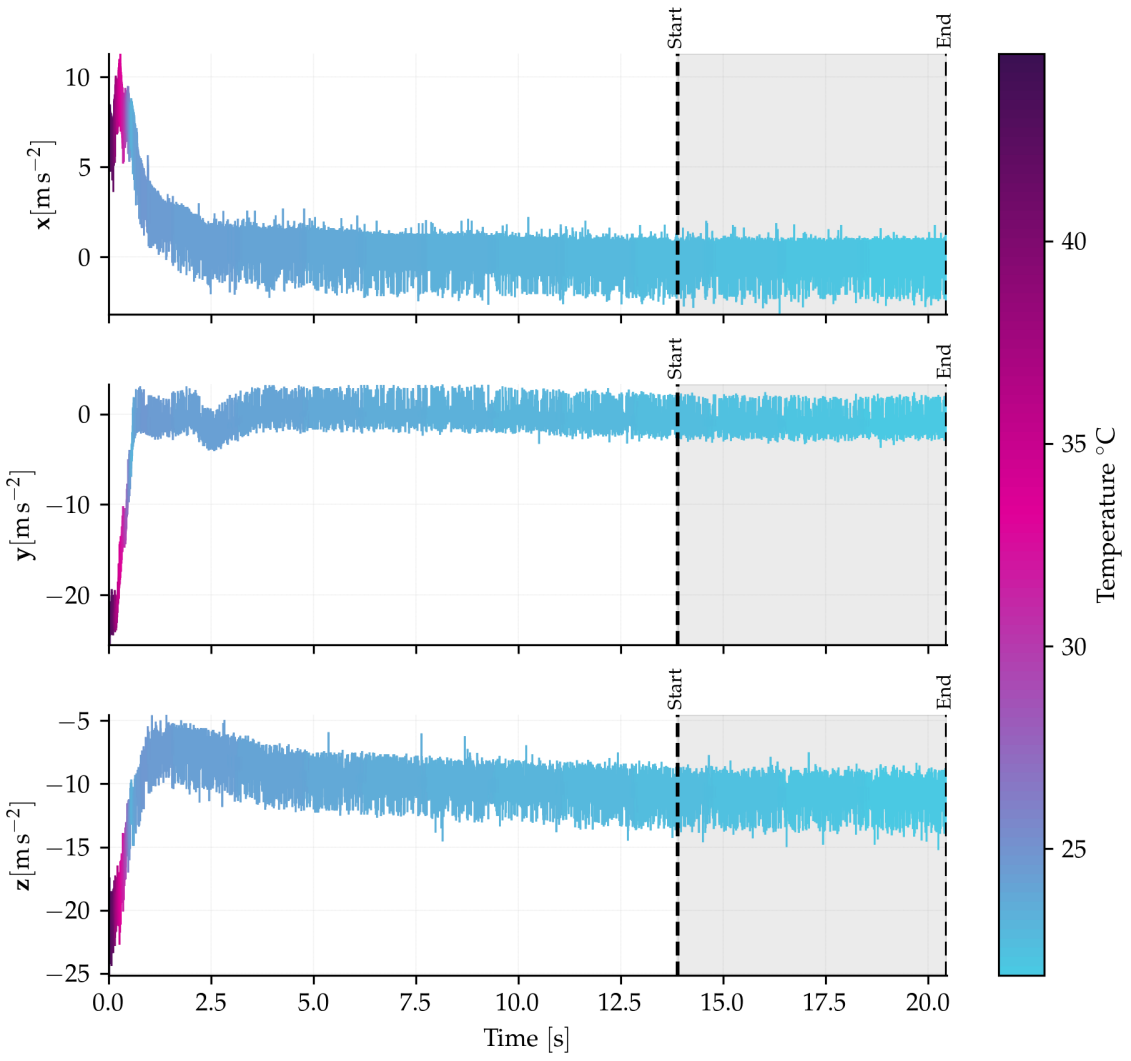
Temporal evolution of the calibrated acceleration signals during the bias instability test inside the climatic chamber. The color gradient represents the sensor’s internal temperature, capturing the initial warm-up transient (from ≈45 °C down to 23.4 °C) and the subsequent thermal stabilization. The gray shaded region, delimited by the dashed lines ‘Start’ and ‘End’, indicates the steady-state period (thermal equilibrium) selected for the Allan Variance analysis shown in Figure 16.

To quantify the sensor’s long-term instability, a test was conducted with the accelerometer at rest inside a climatic chamber. Figure 15 shows the temporal evolution of the calibrated measurements. The chamber was initially set at a temperature of 40 °C. The internal temperature of the sensor, which started at 45 °C, and the chamber set point were subsequently adjusted to 20 °C. The sensor stabilized at a temperature of 23.4 °C after approximately 7 h, highlighting the significant thermal inertia of the assembly. This initial high-temperature transient clearly demonstrates the sensor’s temperature dependence and the limitation of the differential interrogation topology in fully compensating for temperature effects across the entire sensor structure.

**Figure 16:**
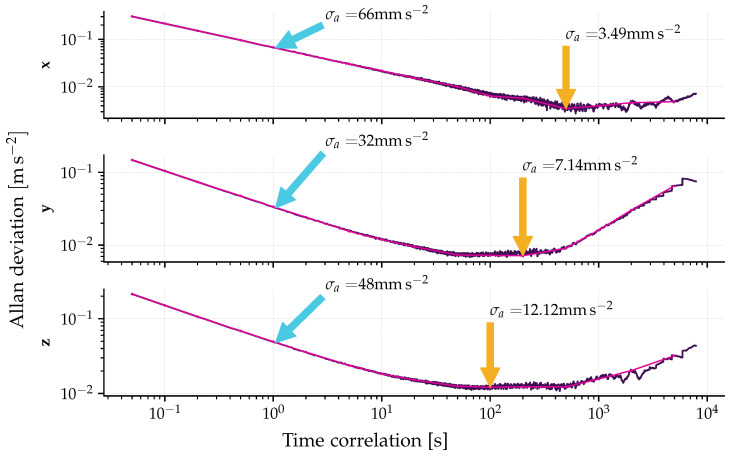
Allan Deviation (ADEV) curves computed for each axis using the steady-state data highlighted in Figure 15. The plots identify the key stochastic noise parameters: the cyan arrows indicate the value at τ=1s (used to determine $\sigma_{VRW}$), while the orange arrows identify the curve’s minimum point, corresponding to the Bias Instability ($\sigma_{b}$) of the sensor.

The Allan Variance analysis was applied to the last six hours of data, corresponding to the period during which the sensor achieved thermal equilibrium. The Allan deviation (ADEV) plot for each axis is presented in Figure 16. From these curves, the VRW and bias instability parameters were extracted, and their values are summarized in Table 2. The observed disparity in the results among the axes is likely attributable to differences in the sensitivity or the positioning of each coupler and optical component. The results, with a bias instability of less than 2 mg (milligravities), classify the sensor as tactical grade, according to the reference [43].

The observed disparity in the results among the axes is likely attributable to minor differences in the coupling ratios and the mounting stress of the specific fiber segments. To contextualize these results within the spectrum of inertial sensor grades, Table 3 presents a comparisom of the accelerometer’s performance with standard industry classifications [44].

As evidenced in Table 3, the achieved bias instability (ranging from 0.54 to 1.87 m$g_{E}$) places the developed sensor within the tactical grade category. This performance level is sufficient for short-duration missions, such as sounding rockets or orbital injection phases, where GNSS denial is temporary and the integration time is limited.

### 4.5. Performance of the Thermal Calibration Model

**Figure 17:**
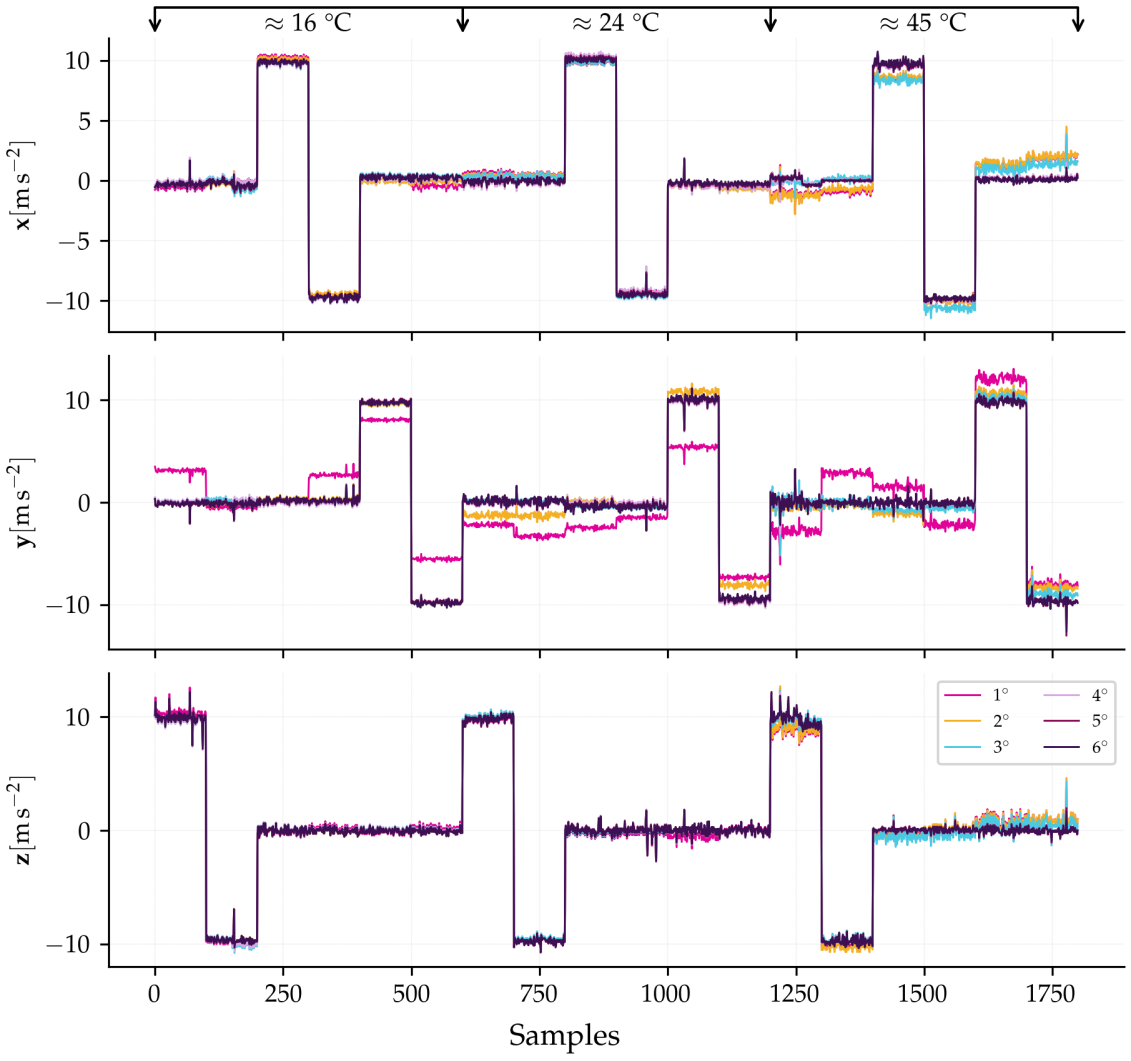
Calibrated measurements with the 3rd-degree polynomial thermal compensation model.

To evaluate the sensor’s performance over an operating temperature range, the calibration procedure was repeated at three temperature levels. The data in Figure 11 are concatenated in a single dataset where the first 600 points represent the data collected at ≈16 °C, the next 600 points represent the data collected in ≈24 °C, and the last 600 points represent the data collected in ≈45 °C. This new dataset is used to estimate the parameters of the polynomial model proposed in Equation (28). Figure 17 displays the data calibrated with the thermal model. The legend indicates the polynomial degree used to model the thermal dependence of the sensor, i.e., represent the term *n* on Equation (30). It is noteworthy that, regardless of the operating temperature (indicated by distinct colors), the sensor outputs converge to the reference values, validating the effectiveness of the compensation method.

**Figure 18:**
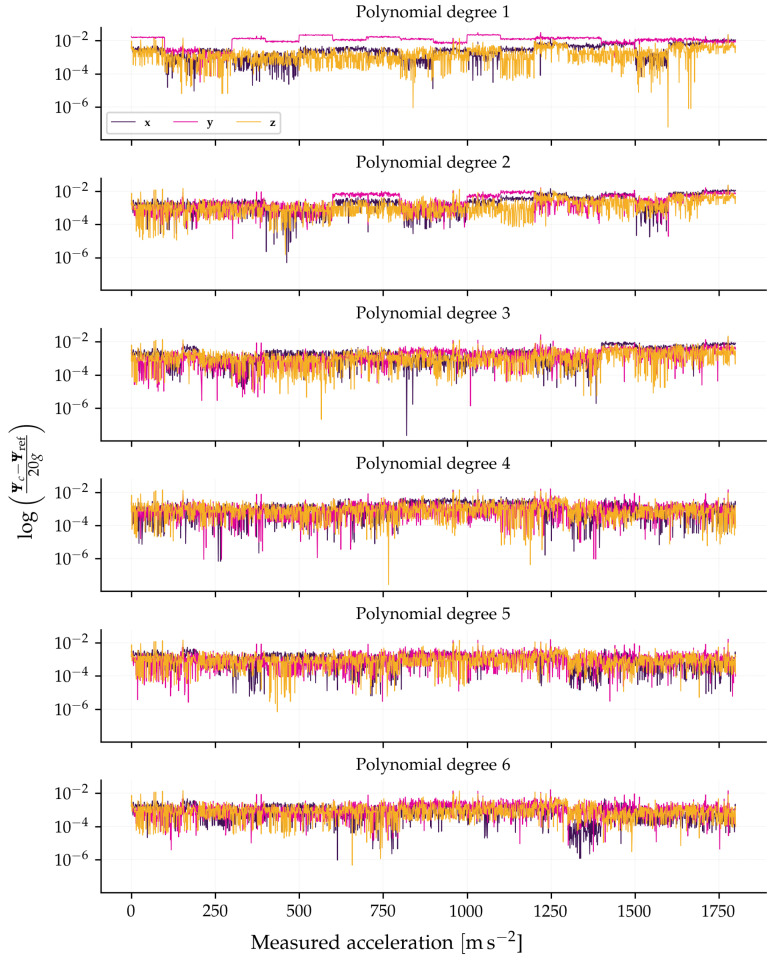
Normalized residual errors as a function of the measured acceleration for different degrees of the polynomial thermal compensation model.

The residual nonlinearity of the sensor was quantified by analyzing the model errors for different polynomial degrees. Figure 18 shows the errors normalized by the full-scale range (20 $g_{E}$). Table 4 presents the root mean square error (RMSE) value of these errors. A drastic reduction in error is observed when moving from the linear model (degree 1) to the quadratic model (degree 2), especially on the y-axis. The third-degree model offers an additional improvement, while higher degrees (4 to 6) do not show significant gains, indicating that the degree-3 model is the most efficient for describing the sensor’s thermal behavior.

The nonlinearity of a sensor quantifies the deviation of the provided measurement from an ideal reference model. To characterize and correct this behavior, the residual errors of the polynomial models are used to quantify the unmodeled nonlinearity. Figure 18 presents these residuals normalized by the full-scale range for models of degree 1 to 6 using Equation (32).

(32) $$e=\frac{\left|\Psi_{c}-\Psi_{\mathrm{ref}}\right|}{20g}.$$

The extraction of a single value to represent the nonlinearity from this noisy data is performed by calculating a robust statistical metric, such as the RMSE, on the residuals of the lowest-degree model that effectively removes systematic trends (in this case, the polynomial of degree 2 or 3). This RMSE value then represents the magnitude of the system’s residual nonlinearity, overcoming the limitation of using the maximum absolute deviation, which would be susceptible to noise outliers.

As discussed in Section 3.6.2, the necessity for a model of order higher than one (linear) is justified by the aggregate behavior of the optoelectronic chain. While the thermal response of the FBG sensor itself is dominated by the linear thermo-optic coefficient of silica over this range, the interrogation system introduces non-linear dependencies.

Specifically, the SLD exhibits a temperature-dependent wavelength drift and spectral shape variation. Since the measurement principle relies on the convolution of the source spectrum with the FBG reflection profiles, any relative spectral shift creates a non-linear variation in the integrated optical power. Furthermore, the splitting ratio of the optical couplers and the responsivity of the photodetectors also exhibit thermal sensitivities. The second and third-order terms in Equation (28) therefore act as lumped parameters to compensate for these combined systemic non-linearities, which cannot be corrected by a simple bias and scale factor adjustment.

Finally, Table 5 presents a comparison between the proposed accelerometer and state-of-the-art FBG-based sensors reported in recent literature. While the referenced works achieve high sensitivities—often exceeding 1000 pm/$g_{E}$—they are predominantly designed for seismic and structural monitoring, operating in AC frequency ranges (>0 Hz) and relying on commercial spectral interrogators. In contrast, the sensor developed in this work focuses on tactical navigation requirements. It employs an embedded differential intensity interrogation system that extends the frequency response down to DC (0 Hz). Furthermore, as highlighted in the table, the primary performance differentiator of the proposed architecture is the bias stability (below 2 m$g_{E}$), demonstrating its suitability for inertial navigation systems where long-term stability and SWaP optimization are more critical than absolute sensitivity.

## 5. Conclusions

This work presented the design, calibration, and performance characterization of a triaxial accelerometer based on FBGs with differential interrogation. A comprehensive calibration methodology, which includes a polynomial model for compensating thermal effects, was proposed and experimentally validated.

The results demonstrate that the sensor has tactical-grade performance, with a bias instability of less than $1.9\;mg_{E}$ for all axes, validating the optomechanical architecture and the low-noise conditioning circuit. The main contribution of this work is the implementation and validation of a calibration model that efficiently corrects bias and scale factor errors induced by temperature variations. It was demonstrated that a third-order polynomial model is sufficient to reduce residual errors to a level dominated by the sensor’s intrinsic noise, ensuring the accelerometer’s accuracy over a wide range of operating temperatures. The obtained results qualify the sensor for applications in inertial navigation systems under different thermal conditions.

The linearity analysis, conducted via spectral convolution, revealed that the sensor maintains a predictable response within the tested range of 180 m/$s^{2}$, with a maximum non-linearity error of approximately 6.7% FS for the *x*-axis. It was observed that the *z*-axis presented superior linear stability compared to the others. This behavior is primarily attributed to the non-identical spectral profiles (bandwidth, reflectivity, and SLSR) of the six inscribed FBGs, which result in distinct convolution integrals for each sensing segment. Furthermore, the triaxial sensor has potential for mechanical integrity, as the structural operating limit exceeds the current optical interrogation range.

As future work, the following are suggested: the miniaturization of the optical and electronic system; the integration of a dedicated higher-resolution ADC; and the performance of dynamic tests to characterize the device’s frequency response.

## Figures and Tables

**Table 1:** Measured power values at rest and conditioning circuit components for each channel.

	Optical Power	$R^{\mathrm{tr}}$		$C^{\mathrm{tr}}$	
		Calculated	Used	Calculated	Used $\left(f^{\mathrm{tr}}\right)$
99%	11 mW				
1%	101 μW	17.5 kΩ	16 kΩ	7.8 μF	$10\;\mathrm{\mu F}\;\left(1\mathrm{Hz}\right)$
$\hat{x}$	730 nW	2.4 MΩ		52.4 nF	$47\;\mathrm{nF}\;\left(1.7\;\mathrm{Hz}\right)$
$\hat{y}$	1.4 μW	1.2 MΩ		104.7 nF	$100\;\mathrm{nF}\;\left(1.3\;\mathrm{Hz}\right)$
$\hat{z}$	2 μW	886 kΩ	820 kΩ	153.2 nF	$100+47\;\mathrm{nF}\;\left(1.3\;\mathrm{Hz}\right)$

**Table 2:** Accelerometer instability performance parameters.

Axis	VRW $\left[m/s/\sqrt{h}\right]$	Bias Instability [${\mathrm{mg}}_{E}$]
x	3.96	0.54
y	1.94	1.10
z	2.87	1.87

**Table 3:** Comparison of the developed sensor’s bias instability with standard inertial navigation performance grades [44].

Performance Grade	Bias Instability	Typical Application
Marine/Navigation	<$0.03\;mg_{E}$	Submarines, Long-haul Aircraft
Tactical	0.1 – 10.0 m$g_{E}$	UAVs, Short-range Missiles
Automotive/Industrial	>$10.0\;mg_{E}$	Airbags, Suspension Control
This Work	<$1.9\;mg_{E}$	Sounding Rockets/Low-LEO

**Table 4:** Normalized RMSE with respect to the degree of the polynomial model to thermal compensation.

Degree of Poly	x	y	z
1	4 × 10 ^−3^	3 × 10 ^−3^	2 × 10 ^−3^
2	3 × 10 ^−3^	4 × 10 ^−3^	1 × 10 ^−3^
3	2 × 10 ^−3^	1 × 10 ^−3^	1 × 10 ^−3^
4	6 × 10 ^−4^	6 × 10 ^−4^	6 × 10 ^−4^
5	5 × 10 ^−4^	7 × 10 ^−4^	6 × 10 ^−4^
6	4 × 10 ^−4^	7 × 10 ^−4^	6 × 10 ^−4^

**Table 5:** Comparison of the proposed accelerometer performance with state-of-the-art FBG-based sensors reported in recent literature.

Reference	Topology	Interrogation	Sensitivity	Freq. Range	Key Feature/Focus
Zhou et al. [24]	Multi-core Fiber	Micron Optics, SM130	355 pm/$g_{E}$	10 Hz to 220 Hz	3D vibration reconstruction
Chen et al. [25]	Thin-cladding Cantilever	Micron Optics, SM130	≈2150 pm/$g_{E}$	0.5 Hz to 30Hz	High sensitivity (Seismic)
Qiu et al. [22]	Dual-Mass	Beijing Weiyun, MWYFBG-CS800	1194 pm/$g_{E}$	1 Hz to 40 Hz	Infrastructure monitoring
Reghuprasad et al. [45]	Cantilever beam	Micron Optics, SI155	433.7 pm/$g_{E}$	5 Hz to 55 Hz	Seismic monitoring
Zhang et al. [21]	Cross-Diaphragm	OF demodulator	590 pm/$g_{E}$	0.1 Hz to 50 Hz	Seismic monitoring
Wang et al. [46]	Cantilever beam	OF FBG Demodulator	1613.3 pm/$g_{E}$	43 Hz (ressonace)	Cable force monitoring
Qiu et al. [12]	Multi-flexible beam	OF demodulator	590 pm/$g_{E}$	0.05 Hz to 80 Hz	Seismic monitoring
Liu et al. [13]	Integrated Single-Mass	Micron Optics, SI155	1025 pm/$g_{E}$	20 Hz to 205 Hz	Low cross-axis error
Cao et al. [9]	Integrated Single-Mass	Ibsen, IMON 512	56.5 pm/$g_{E}$	30 Hz to 80 Hz	Structural health monitoring
Velázquez-Carreón et al. [11]	Integrated Single-Mass	FBG demodulator (unspecified)	1730 pm/$g_{E}$	1 Hz to 20 Hz	Low-frequency
This Work	Single Seismic Mass	Diff. Intensity (embedded)	112 $pm/g_{E}$ (differential)	DC—440 Hz	Thermal Bias Stability (<2 $mg_{E}$)

Note: The sensitivity units differ due to interrogation methods (wavelength shift vs. intensity voltage output), but the key performance differentiator for navigation is the bias stability.

## Data Availability

The data are available on https://doi.org/10.5281/zenodo.18825983, accessed on 16 January 2026.
